# Association of Estradiol and Visceral Fat With Structural Brain Networks and Memory Performance in Adults

**DOI:** 10.1001/jamanetworkopen.2019.6126

**Published:** 2019-06-21

**Authors:** Rachel G. Zsido, Matthias Heinrich, George M. Slavich, Frauke Beyer, Shahrzad Kharabian Masouleh, Juergen Kratzsch, Matthias Raschpichler, Karsten Mueller, Ulrike Scharrer, Markus Löffler, Matthias L. Schroeter, Michael Stumvoll, Arno Villringer, A. Veronica Witte, Julia Sacher

**Affiliations:** 1 Department of Neurology, Max Planck Institute for Human Cognitive and Brain Sciences, Leipzig, Germany; 2 Emotion Neuroimaging Lab, Max Planck Institute for Human Cognitive and Brain Sciences, Leipzig, Germany; 3 Cousins Center for Psychoneuroimmunology, Department of Psychiatry and Biobehavioral Sciences, University of California, Los Angeles; 4 Subproject A1, Collaborative Research Centre 1052 “Obesity Mechanisms,” University of Leipzig, Leipzig, Germany; 5 Institute of Laboratory Medicine, Clinical Chemistry and Molecular Diagnostics, University of Leipzig, Leipzig, Germany; 6 Heart Center Leipzig, Department of Cardiac Surgery, Leipzig, Germany; 7 Integrated Research and Treatment Center (IFB) Adiposity Diseases Faculty of Medicine, University of Leipzig, Leipzig, Germany; 8 Clinic for Cognitive Neurology, University of Leipzig, Leipzig, Germany; 9 Institute for Medical Informatics, Statistics, and Epidemiology (IMISE), University of Leipzig, Leipzig, Germany; 10 Leipzig Research Center for Civilization Diseases (LIFE), University of Leipzig, Leipzig, Germany

## Abstract

**Importance:**

Changes in estradiol during aging are associated with increased dementia risk. It remains unclear how estradiol supports cognitive health and whether risk factors, such as midlife obesity, are exacerbated by estrogen loss.

**Objectives:**

To assess whether visceral adipose tissue (VAT) moderates the association between age and brain network structure and to investigate whether estradiol moderates the association between VAT and brain network structure.

**Design, Setting, and Participants:**

Cross-sectional study of data from 974 cognitively healthy adults in Germany who participated in the Health Study of the Leipzig Research Centre for Civilization Diseases, a previously described population-based cohort study. Two moderation analyses were performed, including VAT as the moderator variable between age and brain network structure and estradiol as the moderator variable between VAT and brain network structure. The study was conducted from August 1, 2011, to November 23, 2014. Analyses were conducted from August 2017 to September 2018.

**Exposures:**

Serum estradiol levels from fasting blood and visceral adipose tissue volume from T1-weighted magnetic resonance imaging (MRI).

**Main Outcomes and Measures:**

Brain network covariance (individual loading on structural network derived from T1-weighted MRI) and memory performance (composite score from the Consortium to Establish a Registry for Alzheimer Disease [CERAD] verbal episodic memory test on learning [score range, 0-30], recall [score range, 0-10], and recognition [score range, 0-20]).

**Results:**

Final analyses included data from 473 women (mean [SD] age, 50.10 [15.63] years) and 501 men (mean [SD] age, 51.24 [15.67] years). Visceral adipose tissue was associated with an exacerbation of the negative association of aging with network covariance for women (interaction term β = −0.02; 95% bias-corrected bootstrap CI, −0.03 to −0.01; *P* = .001) and men (interaction term β = −0.02; 95% bias-corrected bootstrap CI, −0.03 to −0.01; *P* < .001). Estradiol level was associated with a reduction in the negative association of VAT with network covariance in women (interaction term β = 0.63; 95% bias-corrected bootstrap CI, 0.14-1.12; *P* = .01), with no significant association in men. In the female midlife subgroup (age range, 35-55 years, when menopause transition occurs), low estradiol levels were associated with lower memory network covariance (Cohen *d* = 0.61; *t*_80_ = 2.76; *P* = .007) and worse memory performance (Cohen *d* = 0.63; *t*_76_ = 2.76; *P* = .007).

**Conclusions and Relevance:**

This study reports a novel association between VAT, estradiol, and structural brain networks as a potential mechanism underlying cognitive decline in women. These findings appear to highlight the need for sex-specific strategies, including VAT and hormonal screening during midlife, to support healthy cognitive aging.

## Key Points

**Question:**

Does estradiol mitigate the negative association of visceral fat with structural brain networks and cognitive health?

**Findings:**

In this cross-sectional study of a German population-based cohort of 974 adults, higher estradiol levels were associated with increased structural brain network covariance and a reduction in the negative association of visceral fat with network covariance, but only for women. In women, higher estradiol levels were associated with better structural network covariance and cognitive performance during midlife.

**Meaning:**

Assessing visceral adipose tissue and hormone profiles, particularly in women during midlife, may be essential for promoting a healthy brain aging trajectory.

## Teaser

This cross-sectional study of a German population-based cohort assesses whether visceral adipose fat moderates the association between age and brain network structure and whether estradiol moderates the association between visceral adipose fat and brain network structure.

## Introduction

Neuroimaging evidence suggests an association between age-related brain atrophy, cognitive decline, and obesity.^1^ Emerging data linking obesity to increased cognitive impairment in old age are concerning given that approximately 39% of the world’s adult population are overweight and 13% are obese.^2^ Visceral adipose tissue (VAT) is a known risk factor for vascular and metabolic diseases,^3,4,5^ but the results of some studies^6,7^ suggest that elevated VAT may also impair cognitive function. Behaviorally, increased VAT is associated with reduced verbal memory, attenuated attention, and lower executive function. Expanded VAT is further linked to brain atrophy, such as decreased hippocampal volume, cortical thickness, and total brain volume.^6,8^ Consequently, researchers are starting to view visceral obesity in midlife as a risk factor for dementia and depression in later life^9,10^ independent of type 2 diabetes and cardiovascular comorbidities, with the findings of other studies^11,12^ suggesting an even stronger association in women.

Expanded VAT is associated with systemic inflammatory biomarkers, proinflammatory cytokines,^13,14,15^ and reduced levels of adipocyte-specific proteins with anti-inflammatory properties, for which substantial sex differences are reported.^13,16^ Visceral adipose tissue is also associated with dysregulation of the hypothalamic-pituitary-adrenal (HPA) axis and may contribute to cortisol regeneration^17^ and vulnerability to stress-induced cortisol reactivity in women.^18^ Moreover, if VAT compromises brain structural integrity owing to its association with systemic inflammation and HPA dysregulation, estradiol may have a protective role due to its anti-inflammatory properties and ability to strengthen HPA robustness.^19^ This is in line with menopause transition risk models for neuropsychiatric disorders, which suggest that ovarian hormone fluctuations induce alterations in stress response pathways.^20^ Further support for the moderating role of estradiol stems from the finding that estradiol replacement reduces metabolic syndrome symptoms in estradiol-depleted women.^21,22,23^ Estradiol also has vasodilatory, antiapoptotic, and antioxidative effects, which could help preserve myelin architecture.^24,25,26^ Although VAT and estradiol appear to have opposing roles in association with healthy brain aging, it remains unclear how they interactively alter brain network structure. This has serious implications for female cognitive decline in later life because women are potentially more sensitive to the effects of VAT on cognition.^7^ Moreover, dementia in women is likely influenced by midlife obesity,^27^ a time also characterized by rapid, unstable decreases in estradiol.^28^ To investigate these associations in adults, we require integration of brain, abdominal, hormonal, and cognitive data.

We addressed these issues in a novel way by integrating measures of brain network structure, VAT, sex hormones, and cognitive function in a comprehensive data set of 974 participants (473 women) aged 19 to 79 years. We first characterized a structural brain network that shows accelerated degeneration with aging and, when compromised, has been associated with poor memory performance and vulnerability to unhealthy aging and disease.^29,30^ After validating that this network correlated with memory performance, we explored how associations between VAT, estradiol levels, and structural brain network covariance differ between men and women. To our knowledge, this is the first study to examine how these factors interact to shed light on biological mechanisms underlying cognitive decline. In a secondary analysis, we investigated how estradiol levels are associated with network covariance and memory performance specifically in midlife women (age range, 35-55 years) because this is a crucial transition point when midlife obesity is a recognized risk factor for dementia^27^ and estradiol levels are known to fluctuate during the menopause transition.^28^ Based on the research summarized above linking brain network structure, VAT, estradiol levels, and cognitive function, we hypothesized (1) that VAT would be associated with an amplification of the negative association of age with brain network structure and cognitive health and (2) that estradiol would be associated with a reduction in the negative association of VAT with structural network covariance and cognitive performance in women.

## Methods

### Participants

Data from 1159 participants were taken from a German population-based cohort study, the Health Study of the Leipzig Research Centre for Civilization Diseases (LIFE). Participants provided written informed consent after all procedures were explained. The protocol and informed consent forms were approved by the research ethics board of the University of Leipzig. Study design and assessment information were described previously^31^ (eAppendix 1 in the Supplement). Excluded from analysis were 183 individuals owing to medication intake altering the central nervous system, immunosuppressive medication, previous stroke or other lesions, current diagnosed cancer or cancer treatment during the previous year, head tumors, epilepsy, multiple sclerosis, or Parkinson disease. One individual was excluded because of failure of the macro for fat tissue segmentation and another individual owing to missing anthropometric data needed for VAT normalization. In total, 974 participants were included in the final analyses, all of whom were confirmed to not have dementia during a neuropsychological assessment, including the Mini-Mental State Examination, by a trained study physician. Dates of the original cohort study were August 1, 2011, to November 23, 2014. Analyses were conducted from August 2017 to September 2018. This study followed the Strengthening the Reporting of Observational Studies in Epidemiology (STROBE) reporting guideline.^32^

### Brain and Abdomen Imaging

Magnetic resonance imaging (MRI) data were acquired on a 3-T imaging system (Magnetom Verio; Siemens). It was equipped with a 32-channel head array coil and body coil for the transmit-receive coil of abdominal scans.

### Abdominal Data Acquisition and Analysis

Magnetic resonance imaging was performed using an axial T1-weighted fast spin-echo technique with the following variables: echo time of 18 milliseconds per repetition time of 520 milliseconds, echo train length of 7; slice thickness of 5 mm, 5 mm between slices; scanning matrix of 320 × 306 pixels (no partial Fourier); and field of view of 500 mm ×375 mm, final voxel size of 1.6 mm ×1.6 mm ×5.0 mm, water saturation. Nine slices under and 10 slices above the umbilicus, diaphragm excluded, were segmented. The abdominal fat tissue segmentation was graphically evaluated using a macro to distinguish visceral or subcutaneous fat semiautomatically in ImageJ (https://imagej.nih.gov/ij/download/) by 4 raters (M.H., M.R., U.S., and a nonauthor) for accuracy. The results were inspected visually (slice by slice, identifying misclassified fat and nonfat voxels) and corrected for minor voxel misclassifications manually in 897 participants (eAppendix 2 in the Supplement).

### Brain Data Acquisition and Analysis

We collected a 3-dimensional magnetization-prepared rapid acquisition with gradient echo (MPRAGE) sequence using the Alzheimer Disease Neuroimaging Initiative (ADNI) standard protocol with the following variables: inversion time of 900 milliseconds, repetition time of 2300 milliseconds, echo time of 2.98 milliseconds, flip angle of 9°, band width of 240 Hz per pixel, image matrix of 256 × 240 pixels, 176 partitions, field of view of 256 × 240 × 176 mm^3^, sagittal orientation, 1 average. Voxel size was 1 × 1 × 1 mm^3^, with no interpolation.^33,34^ We processed structural T1-weighted data with FSL-VBM (FSL, version 5.0.9), an optimized voxel-based morphometry protocol using FMRIB Software Library (FSL) tools. All structural images were brain extracted, gray matter segmented, and registered to the Montreal Neurological Institute (MNI) 152 standard space using nonlinear registration. A symmetric study-specific gray matter template was built from images of the study population. All native gray matter images were nonlinearly registered to this study-specific template and “modulated” to correct for local expansion or contraction due to the nonlinear component of the spatial transformation. Modulated images were smoothed with an isotropic gaussian kernel with a sigma of 4 mm (approximately 9.4-mm full width at half maximum). Before FSL-VBM processing, volumes were masked by the full brain–segmented volume output from FreeSurfer (FreeSurfer, version 5.3.0) to exclude nonbrain compartments. Brain structural information was derived from vertexwise cortical thickness, and surface area was calculated in FreeSurfer by an automated surface reconstruction scheme. We inspected all surface reconstructions for misplaced boundaries in FreeView (implemented in FreeSurfer) and manually corrected 142 cases. Cortical thickness and surface area maps were sampled from participant space to the common FsAverage template (163 842 vertices) and smoothed with a surface full width at half maximum of 10 mm. Linked independent component analysis was then applied to measures of gray matter volume, cortical thickness, and pial area (http://fsl.fmrib.ox.ac.uk/fsl/fslwiki/FLICA), decomposing the data into 70 independent components. For each component, we calculated an individual course per participant, further indicated as network covariance,^35,36^ which reflects individual loading on the brain network.

### Memory Testing

We used the 10-word verbal episodic memory test from the neuropsychological test battery of the Consortium to Establish a Registry for Alzheimer Disease (CERAD),^37^ from which we calculated a composite score per participant according to previous studies^30,38,39,40^ (eAppendix 3 in the Supplement). The composite score was from the CERAD verbal episodic memory test on learning (score range, 0-30), recall (score range, 0-10), and recognition (score range, 0-20). To assess performance independent of educational achievement, all memory performance scores used are unstandardized education residuals.

### Hormone Measurement

Serum estradiol levels from fasting blood were assessed in a subsample of 390 participants (181 women). Estradiol level was measured by electrochemiluminescence immunoassay (ECLIA) (Cobas; Roche), with a sensitivity of 5.01 pg/mL and representative interassay coefficients of variation of 4.4% to 9.9% for the range of 85 to 110 pg/mL and 2.7% to 5.6% for the range of 501 to 555 pg/mL (to convert estradiol level to picomoles per liter, multiply by 3.671).

### Statistical Analysis

Statistical analyses were performed using SPSS Statistics 24 (IBM) and R 3.3.2 (R Foundation). Visceral adipose tissue values were height standardized and log-transformed. Estradiol levels were log-transformed. We performed a linear regression with memory network covariance as the independent variable and memory performance as the dependent variable, controlling for age. To investigate VAT accumulation rates, we generated regression models for women and men separately using age as the independent variable and VAT as the dependent variable for each 1-year age bin. To assess best fit, we compared *R*^2^ values of linear, quadratic, and polynomial fit of third degree. To examine sex interactions in the association between VAT and memory network covariance, we performed a multiple linear regression with VAT as the independent variable and network covariance as the dependent variable. To assess the association between estradiol level and this network independent of age, we performed a linear regression with estradiol level as the independent variable and network covariance as the dependent variable using unstandardized age residuals of network covariance.

Moderation analyses were conducted separately per sex with the PROCESS macro (SAS Institute), a modeling program using an ordinary least squares–based path analytical framework to test for direct and indirect associations.^41^ We tested regression pathways in a moderation model (first model) (PROCESS, version 3.0) (eFigure 1 in the Supplement). Variables were mean centered before analyses. A 95% bias-corrected bootstrap CI (BBCI), excluding zero and based on 10 000 bootstrap samples, was considered to be a robust result.^41^ In the first model, we assessed significance and stability of the interaction of VAT and age in association with network covariance by defining age as the independent variable, network covariance as the outcome variable, and VAT as the moderator variable. In the second model, we defined VAT as the independent variable, memory network covariance as the outcome variable, and estradiol as the moderator variable.

Finally, we assessed a subgroup of 82 women in midlife (age range, 35-55 years, when menopause transition occurs). In these women, we performed an estradiol-level median split and compared the mean differences in memory network covariance and memory performance using independent-samples *t* tests. All testing was 2 sided, and *P* < .05 was considered statistically significant.

## Results

In total, 974 participants were included in analyses (Table). The female sample (n = 473) had a mean (SD) age of 50.10 (15.63) years (age range, 20-78 years), and the male sample (n = 501) had a mean (SD) age of 51.24 (15.67) years (age range, 19-79) years.

**Table.** Study Population Demographic Characteristics

Variable	All	Women	Men	*P* Value
Age, y				
No. of participants	974	473	501	.26
Mean (SD)	50.69 (15.65)	50.10 (15.63)	51.24 (15.67)	
BMI				
No. of participants	974	473	501	<.001
Mean (SD)	26.47 (4.62)	25.87 (5.13)	27.04 (4.00)	
VAT, cm^3^				
No. of participants	974	473	501	<.001
Mean (SD)	2188.40 (1462.27)	1569.93 (1059.40)	2772.30 (1548.57)	
Estradiol, pg/mL				
No. of participants	390	181	209	<.001
Mean (SD)	40.76 (73.68)	61.84 (103.89)	22.50 (9.61)	

Abbreviations: BMI, body mass index (calculated as weight in kilograms divided by height in meters squared); VAT, visceral adipose fat tissue.

SI conversion factor: To convert estradiol level to picomoles per liter, multiply by 3.671.

### Structural Brain Network Covariance and Memory Performance

**Figure 1.**
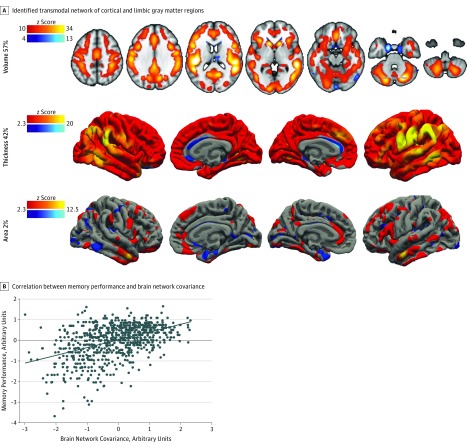
Brain Network Covariance and Memory Performance Color bars indicate *z*-scored positive (red/yellow) or negative (blue/light-blue) covariations within the network.

We replicated a structural network linked to memory performance and cognitive decline^29^ (Figure 1A, eAppendix 4 in the Supplement for network reproducibility analyses, and eFigure 2 in the Supplement for other networks). The network explains 17% of total variance in the imaging data, revealing a transmodal network of cortical and limbic gray matter regions. The network is composed of 57% gray matter volume (explaining 20% of total gray matter volume variance), 42% cortical thickness (explaining 29% of total cortical thickness variance), and 2% pial area (explaining 1% of total pial area variance). Higher individual structural covariance was associated with better memory performance (adjusted *R*^2^ = 0.21; β = 0.46; *P* < .001) (Figure 1B). Data indicate an expected ceiling effect for memory performance in a healthy population.^42^

### Sex Differences in VAT Volume Across Age

**Figure 2.**
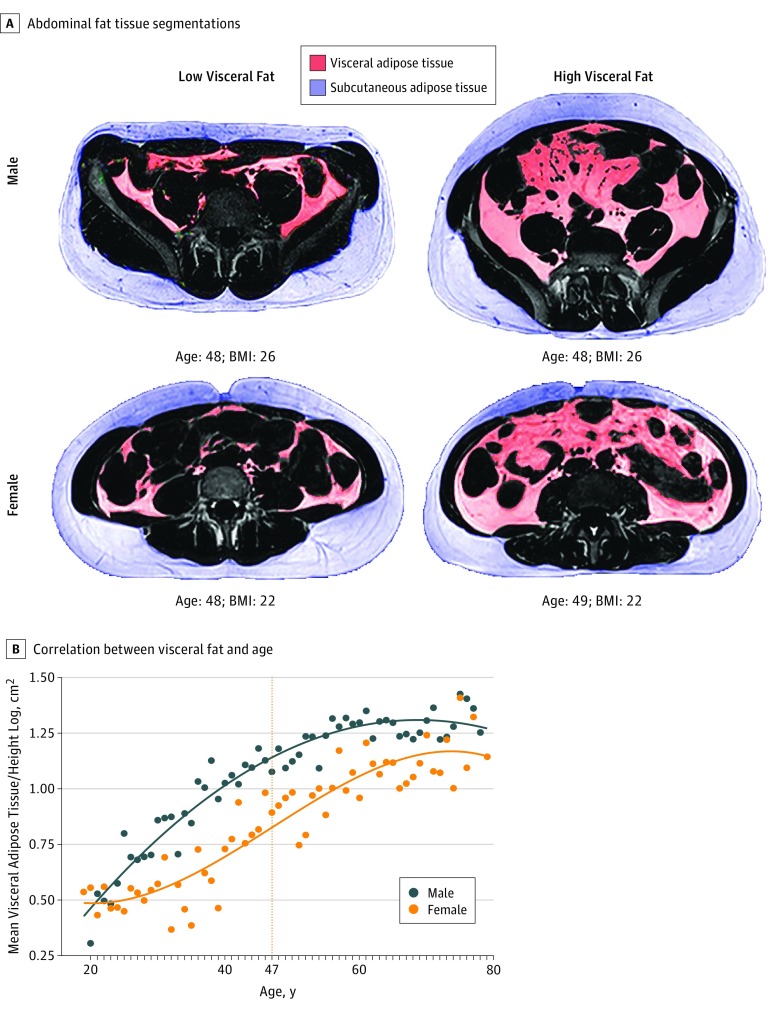
Sex Differences in the Association Between Visceral Adipose Tissue and Age A, Representative visceral adipose tissue segmentations in 2 women and 2 men. Participants of the same sex, age, and body mass index (BMI; calculated as weight in kilograms divided by height in meters squared) can have vastly different fat distribution profiles. B, Nonlinear correlation between visceral adipose tissue and age in women and men. For women, the dashed line shows the inflection point.

We obtained VAT volume information by segmenting visceral from subcutaneous fat in abdominal MRI (Figure 2A). Men and women displayed different trends in VAT accumulation. For men, a quadratic model revealed the best fit (adjusted *R*^2^ = 0.93; *P* < .001) compared with the linear model (*F*_1,58_ = 101.56; *P* < .001) (Figure 2B). The quadratic function showed a concave progression with a steady slope decrease. For women, a polynomial model of third degree showed the best fit (adjusted *R*^2^ = 0.85; *P* < .001) compared with the linear model (*F*_1,59_ = 4.25; *P* = .02) and quadratic model (*F*_2,58_ = 7.74; *P* = .007). The cubic curve progression began convex and became concave at the inflection point of 47 years. Therefore, men in this sample had the highest VAT to age ratio at an earlier age, and this decreased with age; in contrast, women had the highest VAT to age ratio during midlife.

### Sex Differences in VAT and Memory Network Association

**Figure 3.**
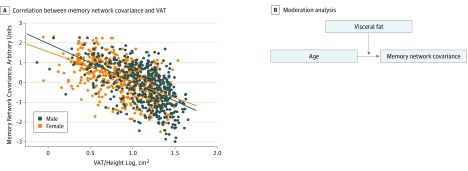
Visceral Adipose Tissue (VAT) and Structural Network Covariance A, The plot shows simple slopes of VAT and structural network covariance for each sex, which differ significantly. The VAT values were height standardized and log-transformed. B, Moderation analysis of interaction of VAT and age on network covariance in both sexes.

We observed an interaction of VAT and sex on memory network covariance (*F*_3_ = 163.37; adjusted *R*^2^ = 0.33; *P* = .02): men showed a stronger negative association between VAT and network covariance (adjusted *R*^2^ = 0.33; β = −0.57; *P* < .001) than women (adjusted *R*^2^ = 0.29; β = −0.54; *P* < .001) (Figure 3A). We conducted a moderation analysis in both sexes (Figure 3B) and found that overall models for men (*F*_3,497_ = 474.43; *R*^2^ = 0.74; *P* < .001) and women (*F*_3,469_ = 322.31; *R*^2^ = 0.67; *P* < .001) were significant. The interaction of VAT and age was significant for women (interaction term β = −0.02; *t*_469_ = 4.07; 95% BBCI, −0.03 to −0.01; *P* = .001) and men (interaction term β = −0.02; *t*_497_ = −4.83; 95% BBCI, −0.03 to −0.01; *P* < .001), suggesting that VAT is a moderator of the association between age and network covariance in both sexes and is associated with an exacerbation of the negative association of aging with memory network covariance.

### Sex Hormones and Memory Network Covariance

**Figure 4.**
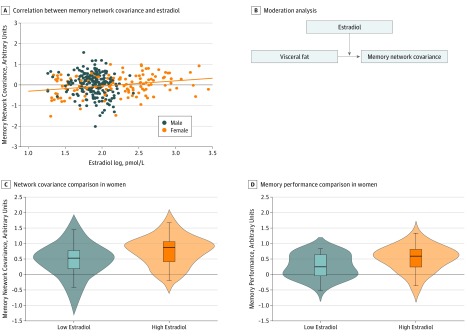
Estradiol and Structural Network Covariance in Women Only A, The plot shows a simple slope of estradiol and structural network covariance. B, Moderation analysis of interaction of visceral adipose tissue and estradiol on network covariance. C and D, The inner box plot shows the median (centers) and interquartile range (borders), with whiskers extending 1.5 times the interquartile range. The width of the shaded area shows the proportion of data located there. To convert estradiol level to picograms per milliliter, divide by 3.671.

Linear regression analysis revealed a significant positive association between estradiol levels and memory network covariance in women after adjustment for age (adjusted *R*^2^ = 0.07; *P* = .002) (Figure 4A). In the next set of moderation analyses (Figure 4B), we again found that overall models were significant for both men (*F*_3,205_ = 30.13; *R*^2^ = 0.31; *P* < .001) and women (*F*_3,177_ = 19.60; *R*^2^ = 0.25; *P* < .001). However, the interaction of VAT and estradiol level was significant only for women (interaction term β = 0.63; 95% BBCI, 0.14-1.12; *t*_177_ = 2.52; *P* = .01), suggesting that estradiol is a moderator of the association between VAT and network covariance in women and is associated with a mitigation of the negative association of VAT with memory network covariance.

Finally, in the female subgroup (age range, 35-55 years), the low estradiol and high estradiol groups did not differ by age or VAT. However, low estradiol level was associated with lower memory network covariance (Cohen *d* = 0.61; *t*_80_ = 2.76; *P* = .007) (Figure 4C) and worse memory performance (Cohen *d* = 0.63; *t*_76_ = 2.76; *P* = .007) (Figure 4D).

## Discussion

To our knowledge, this is the first large, population-based study to investigate associations between structural patterns of brain aging, VAT as a metabolic risk factor for structural brain atrophy, and estradiol levels in adults of a broad age range. Our primary finding is that, while VAT was associated with increased risk for compromised brain network structure and cognitive impairment in both men and women, estradiol level was associated with reducing the negative consequences of VAT in women. Specifically, men had the highest VAT to age ratio at an earlier age; in women during midlife, VAT was associated with accelerated cognitive aging, and estradiol may protect the female brain against these structural patterns of atrophy, particularly during midlife. Our results have important clinical implications for developing sex-specific strategies to support healthy cognitive aging.

To effectively preserve cognitive abilities throughout life, it is imperative to consider sex-specific risk trajectories and identify biological mechanisms that may degrade or protect relevant brain network integrity. Integrating MRI-based VAT in large cross-sectional neuroimaging studies on cognitive function represents a novel approach to identify such a mechanism. We specifically included VAT as the main adiposity measure given the well-established role of VAT in conferring metabolic and inflammatory risk.^3,4,5,13^ Assessing VAT volume by MRI rather than with more conventional and easily accessible anthropometric proxies, such as body mass index or waist to hip ratio, is even more critical given the findings indicating VAT as a unique risk factor for neurodegenerative processes.^10,43,44,45^ Our analysis revealed substantial sex differences not only in VAT accumulation but also in the association between VAT and brain health: while both sexes showed a negative association of VAT with memory network covariance, this association was stronger in men.

When studying a brain network implicated in cognitive decline, it is difficult to control for changes accompanying natural aging. To disentangle the associations of biological aging from the association of VAT with memory network covariance, we tested VAT as a moderator variable in the association between age and the memory network. Our analysis revealed a significant interaction of VAT and age on network covariance, suggesting that VAT is associated with accelerated brain aging. Our findings thus substantially extend prior research showing an association of VAT with brain tissue damage independent of age associations.^46^ While those authors applied age correction in their analysis, the study was conducted in elderly individuals (mean age, 65 years); therefore, the possibility of age as a confounder for the sample could not be excluded.

Although multiple mechanisms may have contributed to the observed sex differences in the association between VAT and network covariance herein, differences in ovarian hormone states across the life span, particularly estrogen fluctuations, have been shown to influence cognitive aging processes.^47,48^ Thus, we investigated the role of estradiol and identified a positive association between estradiol and network covariance in women after age correction. Although the effect size appears small, small associations can suggest strong support for a given phenomenon, particularly if they have substantial cumulative consequences.^49^ Our findings indicate that ovarian aging goes beyond biological age-related brain changes and adds an additional layer to this process. This is important because of the prominent sexual dimorphism in neuropsychiatric disorder rates. A detailed understanding of these processes may provide critical insight into how to address the higher rates of depression^50^ and dementia^51,52^ in women. A subsequent moderation analysis revealed a significant interaction of VAT and estradiol on network covariance in women, suggesting that estradiol is a significant moderator of the VAT-brain association in women. This association could be accounted for by several mechanisms. First, estradiol has anti-inflammatory properties, and systemic inflammation has been shown to occur in response to estradiol depletion in women (eg, after ovariectomy,^53^ natural menopause,^54^ or treatment with the antiestrogenic drug tamoxifen^55^). Second, VAT increases HPA axis dysregulation, which could harm the brain, and estradiol strengthens robustness of HPA activity. Third, estradiol has vasodilatory, antiapoptotic, and antioxidative effects that could have a neuroprotective role and help preserve myelin architecture.^24,25,26^ The present study cannot identify what the mechanism is behind how VAT damages structural networks or how estradiol provides a potential protective association. However, our study results argue for longitudinal studies to assess changes in ovarian hormones, VAT accumulation, cognitive performance, and structural brain networks in women during perimenopause to provide insight into a transition period that may serve as the tipping point into neurodegenerative disease. Moreover, given growing evidence that fluctuations in ovarian hormones and their derived neurosteroids induce HPA axis dysregulation, thereby driving vulnerability to psychosocial stress and neuropsychiatric disease,^20^ a future study could benefit from including markers of HPA axis function in conjunction with psychosocial stress evaluation.

Finally, we investigated the association of estradiol with cognitive health in women during midlife (age range, 35-55 years) because this is when we observed an inflection point in the curve for VAT ratios in women in our cross-sectional sample. It is also the age range when women typically experience rapid estradiol fluctuations and significant estradiol depletion during perimenopause.^28^ We found less healthy patterns of memory network covariance and weaker memory performance in women with lower estradiol levels compared with women with higher estradiol levels during the perimenopausal age range. Because these women were VAT- and age-matched, our findings suggest that associations of ovarian aging with body and brain may extend beyond changes in body composition and brain network structure typically observed during biological aging.^56,57^ This could explain why VAT had less of a negative association with network covariance in women than in men.

### Limitations

It is important to acknowledge the methodological limitations of data-driven neuroimaging analysis, particularly regarding the interpretation of structural brain covariance changes in terms of cellular mechanisms.^29,58^ Specifically, structural MRI is limited to probing information on a macroscopic scale, and extraction of morphological features of interest from MRI remains imperfect, thus making it difficult to disentangle the contributing cellular mechanisms. Despite these constraints, experts^29,58^ still agree that this methodological approach represents a powerful tool to obtain unique insights into human brain organization. By combining this approach with cutting-edge abdominal adiposity imaging and assessment of sex hormone levels**—**and ultimately relating these multimodal measures to memory performance in a large sample**—**we provide a biologically relevant view of the consequences of VAT on brain network structure and cognitive abilities. Furthermore, not all measures herein (estradiol levels and educational achievement) were available for all enrolled participants owing to technical reasons; therefore, some analyses reported could only be conducted in subgroups. We also acknowledge that the CERAD verbal episodic memory test shows ceiling associations in young healthy participants, but the observation that the identified brain network in our sample is associated with memory performance and cognitive decline has also been demonstrated with other cognitive tests.^29^ In addition, brain aging is a multifaceted process; while we applied strict screening criteria to exclude neurodegenerative or cerebrovascular disease and controlled for age and educational achievement, we cannot exclude that other variables could contribute to this process. Inherent to the cross-sectional design, no causal association can be inferred because all reported associations are correlational; therefore, future studies applying within-subject modeling are needed.

## Conclusions

Given the dramatic increase in human life expectancy over the past century, age-related cognitive decline is rapidly becoming one of the biggest health challenges we face.^59,60,61^ Herein, we identified sex-specific risk trajectories of brain and cognitive aging. We provide evidence for a detrimental association of VAT with structural brain networks important for memory and a potential protective association of estradiol with cognitive health in women through maintaining gray matter network integrity. These data underscore the need to consider adipose tissue and hormonal profiles during primary care visits in midlife to support healthy brain aging and maintain cognitive abilities in later life. Our findings highlight the perimenopausal transition as a window of opportunity to prevent accelerated brain aging and neurodegenerative disease development in women.

## Notes

Supplement. **eAppendix 1.** Participant Recruitment **eAppendix 2.** Abdomen: Data Acquisition and Analysis **eAppendix 3.** Memory Testing **eAppendix 4.** Reproducibility of Structural Network From Linked-Independent Component Analysis **eFigure 1.** Conceptual and Statistical Diagram for Moderation Analyses **eFigure 2.** Spatial Maps of Brain Networks **eReferences.**
