# Supplementary material for: Association of Estradiol and Visceral Fat With Structural Brain Networks and Memory Performance in Adults
Source: JAMA Netw Open. 2019 Jun 21;2(6):e196126. doi: 10.1001/jamanetworkopen.2019.6126 (PMC6593958; doi:10.1001/jamanetworkopen.2019.6126)
Supplement: Supplement. — eAppendix 1. Participant Recruitment eAppendix 2. Abdomen: Data Acquisition and Analysis eAppendix 3. Memory Testing eAppendix 4. Reproducibility of Structural Network From Linked-Independent Component Analysis eFigure 1. Conceptual and Statistical Diagram for Moderation Analyses eFigure 2. Spatial Maps of Brain Networks eReferences. [file jamanetwopen-2-e196126-s001.pdf]

Supplementary Online Content

Zsido RG, Heinrich M, Slavich GM, et al. Association of estradiol and visceral fat with structural brain networks and memory performance in adults. *JAMA Netw Open*. 2019;2(6):e196126. doi:10.1001/jamanetworkopen.2019.6126

eAppendix 1. Participant Recruitment

eAppendix 2. Abdomen: Data Acquisition and Analysis

eAppendix 3. Memory Testing

eAppendix 4. Reproducibility of Structural Network From Linked-Independent Component Analysis

eFigure 1. Conceptual and Statistical Diagram for Moderation Analyses

eFigure 2. Spatial Maps of Brain Networks

eReferences.

This supplementary material has been provided by the authors to give readers additional information about their work.

© 2019 Zsido RG et al. *JAMA Network Open*.

eAppendix 1. Participant Recruitment. Participant recruitment is previously reported in detail[1]. Briefly, the LIFE study is a population-based cohort study that examined approximately 10,000 randomly selected participants from Leipzig, Germany. The study was conducted by the Leipzig Research Centre for Civilization Diseases. The baseline examination was conducted from August 2011 to November 2014. All participants underwent a core assessment including questionnaires, structured interviews, physical examinations, and biospecimen collection. A subset of these participants additionally completed MRI-based head (>2400) and abdominal scans (>1000).

The sample compromised of age and gender-stratified sample of Leipzig residents. Address lists of randomly selected citizens were given by the resident's registration office. Citizens were sent an invitation letter (information leaflet about the study, a response form, postage-paid return envelope) and a reminder letter (if no response within four weeks). Non-responders were contacted by phone. Participation rate was 33%.

All participants included in the final analyses completed a neuro-psychological assessment with a trained study physician, including the Mini-Mental State Examination (MMSE) to confirm inclusion of cognitively healthy, non-demented participants. We used a clinical cut-off score of 24[2,3], although we acknowledge that the validity of this cut-off score for dementia is still debated in literature.

© 2019 Zsido RG et al. *JAMA Network Open*.

eAppendix 2. Abdomen: Data Acquisition and Analysis. MR imaging was performed using an axial T1-weighted fast spin-echo technique with the following parameters: TE=18 ms/TR=520 ms, echo train length 7; slice thickness 5 mm, 5-mm gap between slices; scanning matrix 320×306 (no partial Fourier); field of view 500 mm×375mm, final voxel size 1.6 mm×1.6 mm×5.0 mm, water saturation. To avoid breathing artefacts, all participants were asked to hold their breath for 18 seconds, during which 5 slices were recorded. Images were recorded from feet-to-head direction with 5 cm table shift after each acquisition, beginning 10 cm below the umbilicus and finishing in the liver region. All participants were scanned in the supine position with their arms above the abdominal region. We used 20 slices from each participant that included the umbilical region as the center and excluded the diaphragm. We segmented 9 slices under and 10 slices above the umbilicus. This ensured that the umbilical region was included but adipose tissue beyond the diaphragm was excluded in each participant. The graphic evaluation of the abdominal fat tissue was done with ImageJ (https://imagej.nih.gov/ij/download/). We used a macro that quantifies fat pixels semi-automatically using a threshold/histogram algorithm and distinguishes between visceral and subcutaneous adipose tissue, and used manual delineation when necessary. In short, the segmentation algorithm is based on image intensities whereas conventional automated thresholding evaluates fat from non-fat tissue. Using interpolation, the macro calculates adipose tissue volumes as the sum of fat pixels x slice thickness (*ie*, slice+gap). Visceral fat was defined as adipose tissue within the abdominal cavity; subcutaneous fat was defined as adipose tissue between the skin and musculoskeletal tissues. Intramuscular fat was excluded from the analyses. After applying the algorithm, the evaluator visually inspected the results slice-by-slice in order to identify misclassified voxels (fat versus non-fat voxels). Manual corrections for misclassifications were fixed in 897 participants. The most common cases involved myofascial fat tissue being misclassified as subcutaneous fat, and vertebral bodies being misclassified as visceral fat (this is because both have higher intensities in T1-weighted images). To ensure the accuracy of our adipose tissue segmentation method, four different raters applied the segmentation macro on the participants.

© 2019 Zsido RG et al. *JAMA Network Open*.

eAppendix 3. Memory Testing. We assessed participants' memory performance using the neuropsychological test battery of the Consortium to Establish a Registry for Alzheimer's Disease (CERAD)[4]. We focused on the 10-word list for the verbal episodic memory test, from which we calculated three sum scores: "learning" was the number of correctly named words out of three consecutive learning trials, "recall" was the number of correctly recalled words from the list after a delay of approximately 5 minutes (during which participants performed a figure copy task), and "recognition" was the number of correctly recognized words from a list of 20 mixed words presented after the recall trial. All test scores were z-transformed. A composite score for the cognitive domain of memory performance was calculated per participant according to previous studies[5-8] as the mean of the three sub-scores. If a sub-score was missing, the composite score was calculated based on the average of the remaining available sub-scores.

© 2019 Zsido RG et al. *JAMA Network Open*.

eAppendix 4. Reproducibility of Structural Network from Linked-Independent Component Analysis. We visually compared the spatial maps of each component with the structural gray matter network previously linked to memory performance and cognitive decline ([9]; template available at http://www.fmrib.ox.ac.uk/analysis/LIFO+AD+AOS/) and chose the component with the highest accordance. We applied adaptive thresholding to visualize the spatial maps of our network to see where the highest covariations existed (resulting in z values: >14.1247 for positive and <-2.2584 for negative covariations) (see [10] for further details). We then computed voxel-by-voxel spatial cross-correlations with our network and the provided template to ensure the similarity of both spatial maps. Significance of the spatial correlation was calculated using a Monte Carlo approach with randomly generated and smoothed 1000 Gaussian noise images and comparing the strength of our observed correlation with empirically generated null-distribution from 1000 cross-correlations between each of these noise maps and our network (r=0.69 for the raw spatial map, r=0.65 for the thresholded spatial map). Furthermore, we performed a dual regression on the template network with our network using the four-dimensional dataset of gray matter images in a spatial regression against the template network (r=0.74, p <0.001). Finally, we performed a dual regression on our thresholded network and correlated these weights with the weights of the dual regression on the template network (r=0.79, p<0.001). To confirm that our network was the best fit out of the 70 components, we chose the 10 networks based on the elbow in a scree plot of the relative amount of total variance explained per component; beyond explaining the most variance within our imaging data across participants, our network also explained the most age-related variance (R^2_{adj}=0.70, p<0.001) and had the strongest association with memory performance (R^2_{adj}=0.21, p<0.001). Both associations survived correction for multiple comparisons. These analyses confirmed a high comparability of linked-independent component analysis results in this structural network and the previously identified network, indicating reproducibility of results in this method. Spatial maps of the other networks can be viewed in **eFigure 2**.

© 2019 Zsido RG et al. *JAMA Network Open*.

eFigure 1. Conceptual and Statistical Diagram for Moderation Analyses

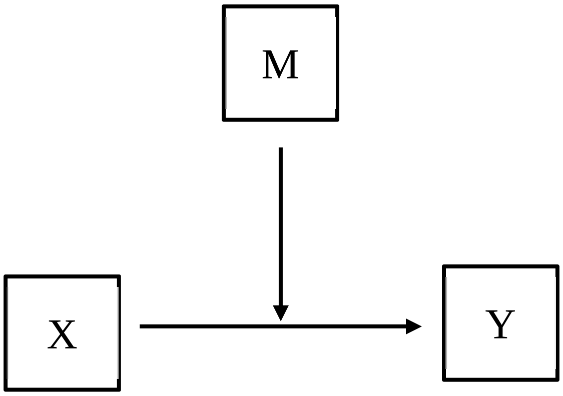

Conceptual Diagram. X is the independent variable. Y is the outcome variable. M is the moderator variable.

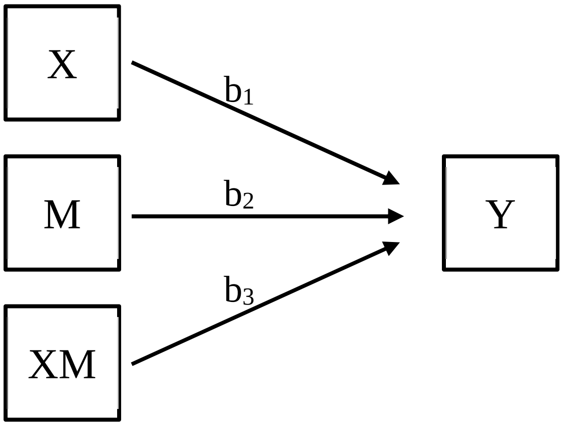

Statistical Diagram. X is the independent variable. Y is the outcome variable. M is the moderator variable. b_1, b_2, b_3 are the beta coefficients.

Conditional effect of X on Y = $b_1 + b_3M$

Diagrams adapted from Hayes AF. *Introduction to mediation, moderation, and conditional process analysis: A regression-based approach.* 2nd ed: Guilford Publications; 2017.

© 2019 Zsido RG et al. *JAMA Network Open*.

eFigure 2. Spatial Maps of Brain Networks

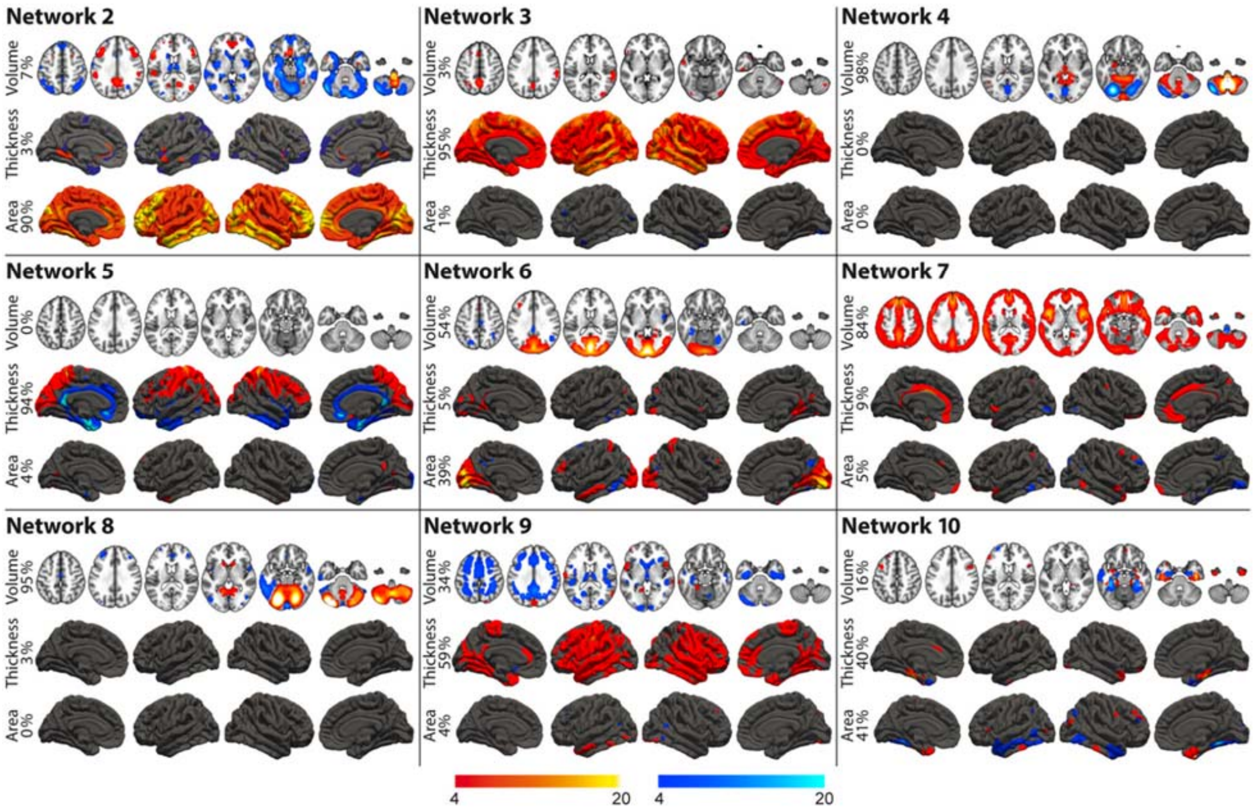

We assessed the spatial maps of the network components resulting from the linked independent component analysis. We chose the top ten networks based on the elbow in a scree plot of the relative amount of total variance explained by each component. The first network explains the most variance and is displayed in Figure 1A. The other nine components are displayed here, along with the contribution of each modality (gray matter volume, cortical thickness, and pial area) to the generation of the network. To confirm specificity of the first network to memory performance (Figure 1B), we assessed the relationship between the other nine networks and memory performance. Our network also explained the most age-related variance (R^2_{adj}=0.70, p<0.001) and had the strongest association with memory performance (R^2_{adj}=0.21, p<0.001). Both associations survived correction for multiple comparisons.

© 2019 Zsido RG et al. *JAMA Network Open*.

References:

1. Loeffler M, Engel C, Ahnert P, et al. The LIFE-Adult-Study: objectives and design of a population-based cohort study with 10,000 deeply phenotyped adults in Germany. *BMC Public Health.* 2015;15(1):691.
2. Kukull WA, Larson EB, Bowen J, et al. The Mini-Mental State Examination score and the clinical diagnosis of dementia. *J Clin Epidemiol.* 1994:47(9):1061–1067.
3. Crum RM, Anthony JC, Bassett SS, et al. Population-based norms for the Mini-Mental State Examination by age and educational level. *JAMA.* 1993:269(18):2386–2391
4. Morris J, Heyman A, Mohs R, et al. The consortium to establish a registry for Alzheimer's disease (CERAD): I. Clinical and neuropsychological assessment of Alzheimer's disease. *Neurology.* 1989.
5. Masouleh SK, Arélin K, Horstmann A, et al. Higher body mass index in older adults is associated with lower gray matter volume: implications for memory performance. *Neurobiol Aging.* 2016;40:1-10.
6. van de Rest O, Geleijnse JM, Kok FJ, et al. Effect of fish oil on cognitive performance in older subjects A randomized, controlled trial. *Neurology*. 2008;71(6):430-438.
7. Witte AV, Kerti L, Hermannstädter HM, et al. Long-Chain Omega-3 Fatty Acids Improve Brain Function and Structure in Older Adults. *Cereb Cortex*. 2014;24(11):3059-3068.
8. Zhang R, Beyer F, Lampe L, et al. White matter microstructural variability mediates the relation between obesity and cognition in healthy adults. *NeuroImage*. 2018;172:239-249.
9. Douaud G, Groves AR, Tamnes CK, et al. A common brain network links development, aging, and vulnerability to disease. *Proc Natl Acad Sci USA.* 2014;111(49):17648-17653.
10. Gorgolewski K, Storkey AJ, Bastin ME, Pernet CR. Adaptive thresholding for reliable topological inference in single subject fMRI analysis. *Front Hum Neurosci.* 2012;6:245.

© 2019 Zsido RG et al. *JAMA Network Open*.
